# A two-step surgery and a multidisciplinary approach in a centenarian patient with an acute presentation of right colon cancer

**DOI:** 10.1186/s12893-020-00708-9

**Published:** 2020-03-18

**Authors:** Chiara Giannotti, Andrea Massobrio, Daniela Cannata, Alessio Nencioni, Fiammetta Monacelli, Alessandra Aprile, Domenico Soriero, Stefano Scabini, Davide Pertile

**Affiliations:** 1 Geriatrics Clinic, Department of Internal Medicine and Medical Specialties (DIMI), University of Genoa, 16132 Genoa, Italy; 2 Oncological Surgery, San Martino Hospital, Polyclinic and Institute for Research and Care, Genoa, Italy; 3 San Martino Hospital, Polyclinic and Institute for Research and Care, Genoa, Italy; 4 Department of Anesthesia and Resuscitation, San Martino Hospital, Polyclinic and Institute for Research and Care, Genoa, Italy

**Keywords:** Colon cancer, Surgery, Cecostomy, Oldest old, Frailty, Comprehensive geriatric assessment

## Abstract

**Background:**

As surgery remains the cornerstone of colorectal cancer (CRC) treatment, the number of older patients presented for colorectal resection is rapidly increasing. Nevertheless, the choice to operate an oldest-old patient still remain challenging and requires a careful assessment of risk to benefit ratio in order to guarantee appropriate surgical strategies and perioperative management.

**Case presentation:**

A centenarian patient, acutely admitted to the emergency department, was diagnosed with an ileus caused by stenosing ascending colon cancer with abnormal distension of the right colon at high risk of perforation. Facing with this complex clinical scenario, a lateral decompressive cecostomy as alternative surgical procedure, was performed in local anesthesia in order to avoid the stressful event of an emergency surgery. Thereafter, the patient was admitted to the surgical ward and followed by a geriatrician who performed a comprehensive geriatric assessment (CGA) and daily clinical evaluations. This integrated plan of care was mainly focused on rehabilitation, nutritional interventions and therapeutic reconciliation, maximizing patient’s clinical conditions and performance status. Then, the second surgical step, the radical colon surgery with curative intent and bowel continuity reestablishment was performed, demonstrating to be feasible and safety also in a very advanced age patient in term of prolonged survival and preservation of an adequate quality of life.

**Conclusions:**

This is the first case-report that illustrates a successful two step surgery for CRC in a centenarian patient thanks to a multidisciplinary based approach, overwhelming the mere concept of chronological age.

## Background

More than 60% of the new diagnoses of colorectal cancer (CRC) are made in patients aged 70 years or older and this estimate is expected to further increase as a result of the ageing process [1]. Surgery represents the first line treatment for colorectal cancer and a growing increase of CRC surgical procedures has been observed, in the old age population, including the “oldest-old” (aged 85 years and more) patients. Age-related anatomic and physiologic changes could affect older adult’s’ ability to cope with an environmental stress such as surgery, potentially leading to higher morbidity and mortality [2]. In line with that, the perioperative management of older surgical patients still remains a challenge and the accurate benefit to clinical ratio for surgery in an oldest-old patient is highly advocated on a [3] multidisciplinary basis to deliver the best clinical care [4].

This case-report illustrates the successful two step surgery for CRC in a centenarian patient with cancer, performed on a highly integrated multidisciplinary basis.

## Case presentation

The patient is a 100-year-old man who entered the emergency department (ER) of the IRCCS Policlinico San Martino, Genoa, Italy for abdominal distension and pain with constipation in the last four days. His abdomen was swollen with a palpable mass in his lower right quadrant.

The medical history included insulin-dependent diabetes mellitus, glaucoma, osteoporosis, osteoarthritis and a laparotomy for cholecystectomy in 2008, and a subsequent open surgical procedure for biliary peritonitis.

The patient’s medical regimen included insulin glargine (6 U/die), ursodeoxycholic acid (300 mg/day), pantoprazole (20 mg/day) and Travoprost drops for glaucoma.

At ER admission the patient’ clinical parameters included a mild temperature of 37.0 °C, a blood pressure of 130/70 mmHg and a pulse rate of 100 beats/min. Blood test results showed an increased white blood cells count of 12.000/mm3 (neutrophils 10.500/mm3), moderate to severe anemia (hemoglobin concentration of 7,8 g/dL; MCV 79,2 fL), platelets 399.000/mm3, C-reactive protein 87,0 mg/dL and low albumin 2.7 g/dL. Renal and liver function, total and direct bilirubin were all within normal levels.

**Fig. 1:**
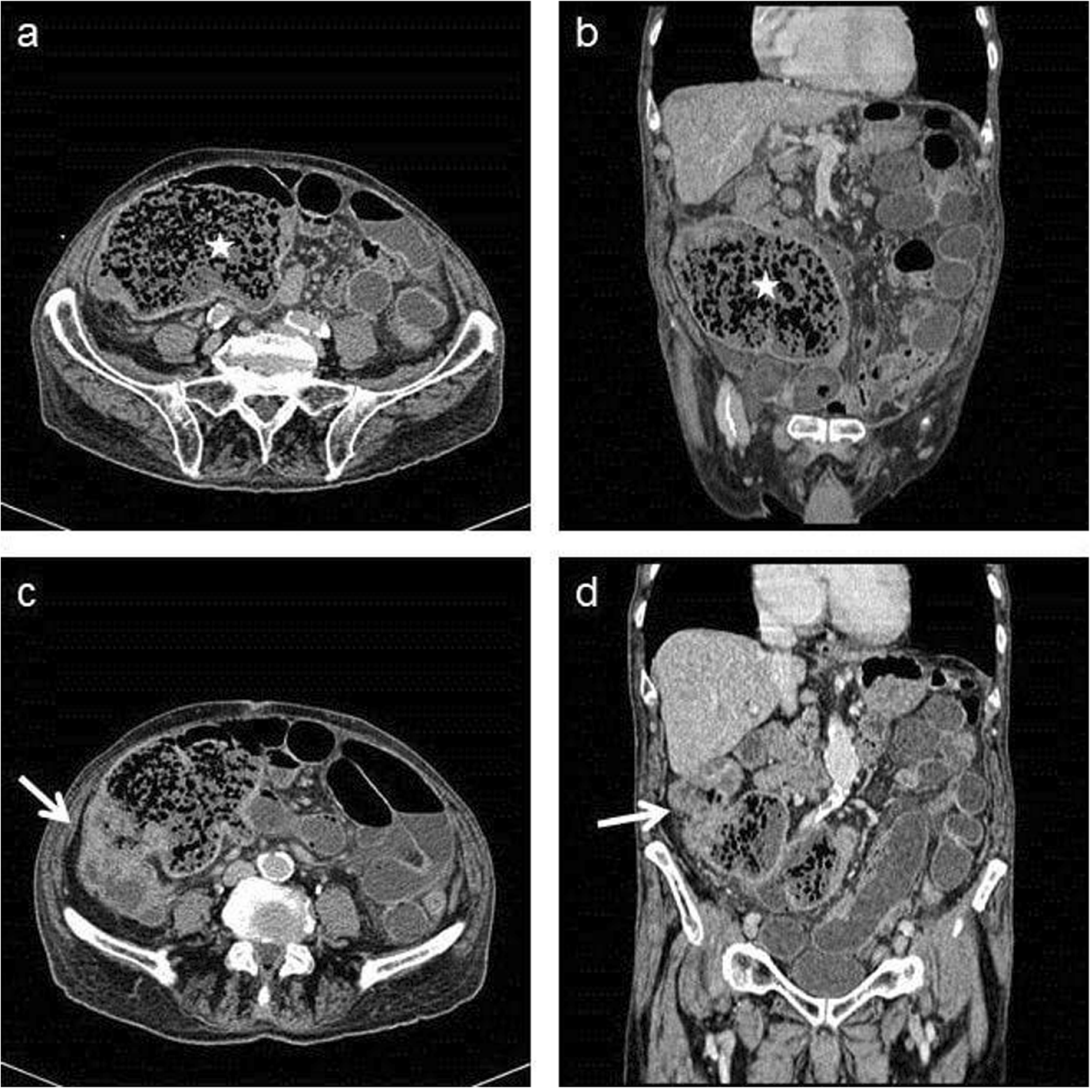
(**a-b**) Contrast-enhanced axial and coronal CT scans show the presence of the distension of the cecum, containing fecaloid material (star). (**c-d**) Contrast-enhanced axial and coronal CT scans show the cancer lesion

Abdominal X-ray showed dilated small bowel segments in the left and middle area of the abdomen with air-fluid levels. An CT scan showed a 6-cm-long, circumferential and stenosing mass in the ascending colon (immediately below hepatic Sg6), hydro-air levels of the small intestine, especially in the ileal area, and the pelvis, the distension of the cecum and of the distal segment of the ascending colon, with a maximum diameter of 9.5 cm, containing fecaloid material (Fig. 1 a-d). There was no radiological sign of metastases in the liver or lungs.

The patient was diagnosed with an ileus caused by stenosing ascending colon cancer with abnormal distension of the right colon at higher visceral risk of perforation. The patient was asked for consent for the emergency surgery consisting of right colectomy and primary anastomosis, as recommended in the current guidelines [5]. However, the patient refused to give his informed consent for surgery and, as an alternative surgical procedure, a lateral decompressive cecostomy without one-stage primary tumor resection during emergency operation was performed in local anesthesia (operating time about 25 min), minimizing the risk for colon perforation with preoperative blood transfusion and intravenous fluid replacement.

Thereafter, the patient was shifted to the surgical ward for post-operative clinical management based on a multidisciplinary team-based approach including a geriatrician who was in charge of the patients’ clinical conditions on a daily basis by virtue of a comprehensive geriatric assessment (CGA). On the basis of the CGA assessment, a series of specific clinical conditions were identified such as malnutrition, hypomobility and the potential drug related iatrogenic syndrome and the appropriate therapeutic interventions were performed, including nutritional oral supplementation, rehabilitation and drug deprescribing (Table 1).

**Table 1:** Comprehensive Geriatric Assessment (CGA) at admission to the surgical ward

*SCREENING TOOL, CGA ASSESSMENT, FRAILTY ASSESSMNT*	*CUT OFF*	*Score*	Target Interventions
*MMSE* [6]	< 24	*21/30*	delirium prevention strategies
*MNA* [7]	< 23	*17/30*	nutrition support
*IADL* [8]	≤ 7	*3/8*	–
*BARTHEL INDEX* [9]	< 50	*90/100*	–
*CIRS SEVERITY* [10]		*1.61/5*	–
*CIRS COMORBIDITY* [10]	> 3	*2/13*	medical comorbidities optimization
*N° OF DRUGS*	≥ 3	*3*	therapeutic reconciliation
*GDS* [11]	≥ 5	*3/15*	*–*
*TINETTI SCALE* [12]	≤ 18	*12/28*	rehabilitation program
*GIJON SCALE* [13]	≥ 10	*9*	family counselling and planning of postoperative discharge needs
*FRAILTY INDEX* [14]	*Fit ≤ 0,08* *0,08 > Pre-frail < 0,25* *Frail ≥ 0,25*	*0.22*	

Abbreviations: *MMSE* Mini Mental State Examination, *MNA* Mini Nutritional Assessment, *IADL* Instrumental Activities of Daily Living, *CIRS* Cumulative Illness Rating Scale, *GDS* Geriatric Depression

The pre morbid patient’s clinical condition showed that he lived independently at home, received appropriate social support, (Gijon scale [13] 7/25), had an initial impairment in both the instrumental and basal activities of daily living (Barthel Index [9] 90/100) in (Instrumental Activities of Daily Living – IADL [8] 3/8) with a faster functional decline experienced in the last two weeks. During the in-hospital stay, the patient had preserved consciousness (Glasgow Coma Scale score of 15/15). The Mini-Mental State Examination [6] was used to screen the cognitive status (MMSE 21/30), while the presence of delirium was assessed daily for 5 consecutive days with the 4AT test [15] (4AT score: 2/12; 3/12; 2/12 at first, third and fifth post-operative day respectively). Non-pharmacological based strategies and interventions were adopted to minimize the risk of incident postoperative delirium targeting sleep disrupted circadian rhythm, visual or hearing deprivation, immobility and dehydration [16].

A mild multimorbidity burden (Cumulative Illness Rate Scale for Geriatrics [10], CIRS comorbidity 2/13 and severity 1.6/5) was assessed. During the in-hospital stay drug deprescribing has led the reduction in the two drugs regimens, minimizing the risk for iatrogenic syndrome Clinical examination was performed on a daily basis with maintained satisfactory cardiopulmonary function and clinical parameters.

A reduced gait speed (0.3 m/sec) with the need of a walker and an increased risk of fall (Tinetti Scale [12] 12/28) were observed during the in hospital stay and a rehabilitation training program was started to recover motor and functional abilities.

During the postoperative in hospital stay, the patient had favorable post-operative outcomes with a prompt canalization of the ostomy that allowed the successful administration of a solid diet the day after surgery.

In particular, the Mini Nutritional Assessment [7] (MNA 17/30) showed mild malnutrition that along with unintentional weight loss, poor appetite and substantial decreased food intake, motivated the request for a nutritional advice with the introduction of a parenteral nutrition, including glucose, lipids and amino acids (Olimel® N4E, 1000 ml, 700 Kcal), associated with the standard diet. Furthermore, in order to prehabilitate the patient for the second surgical step, an oral immunonutrition (Impact® Oral, 237 mL; Nestle) was also administered three times daily in the 5 day before surgery.

After all these clinical interventions, the clinical case was discussed in the disease management team (Surgeon, Oncologist, Geriatrician, Anesthesiologist) and the safety and tolerability of the second surgical step, that included the radical colon surgery with curative intent and bowel recanalization was shared and approved. The patient gave his informed consent to this second step surgical procedure.

The patient underwent midline vertical laparotomy incision extended supra-and-sub-umbilical for 20 cm, under general anesthesia. A right hemicolectomy with ileocolic latero-lateral stapled anastomosis were performed. Intraoperatively, the patient was monitored with bi-spectral index (BIS) and train of four (TOF), in order to adequately modulate the administration of anesthetic drugs. The surgical and anesthesia procedure lasted 35 min and the BIS range underwent a change between 82 (awaked patient) and 30 (asleep), with the BIS value that was never lower than 30, as strongly recommended by recent guidelines dedicated to elders’ anaesthesia and peri-operative care [17–19].

Furthermore, the systolic pressure was maintained stable, with a Mean Arterial Pressure (MAP) > 60 mmHg, and an invasive hemodynamic monitoring (radial catheter) was performed. In order to maintain an adequate body temperature, the patient was heated by infusing liquids through hot-lines and a convective hot air system.

A balanced general anesthesia was used with closed circuit mechanical ventilation and PEEP support. Isoflorane was used as a halogenated gas. Curalization was obtained with Rocuronium and analgesia with Remifentanyl.

Ten minutes before the end of surgery, the patient was given paracetamol 1 g intravenously. This therapy was repeated every eight hours for twenty-four hours, delivering appropriate pain control through an opioid-free analgesia.

During surgery, a nasogastric tube was placed and removed before extubating the patient.

At the end of surgery, the patient was awakened in the operating room and kept in the recovery room for about an hour. Given the stable hemodynamic profile and the adequate consciousness, he was transferred back to the surgical ward.

The patient successfully recovered from this second step surgery without immediate postoperative delirium. A dehiscence of the surgical wound was observed, needing daily medications. From the 4th day after surgery, the patient was administered oral re-feeding and started rehabilitation training program after six days from surgery. Ten days after surgery, the patient was transferred to the Geriatric Clinical ward for clinical continuity of care and extensive rehabilitation.

The histopathological findings of the surgical specimen showed a mucinous (G2) adenocarcinoma with moderate differentiation, with a superficial ulcer, infiltrating the wall up to the sub serosal adipose tissue (pT3), no venous invasion (pV0) and no perineural invasion (pPn0). The surgical margins were all cancer free. A total of 27 lymph nodes were removed, and a single metastasis (1/27 - pN1a- Lymph node ratio: 0.04) was observed. The colon cancer was diagnosed as stage IIIB (pT3, N1a, M0) according to the 7th edition of the International Union Against Cancer TNM classification. Immunostaining for mismatch repair proteins found an immunophenotype with microsatellite instability (MMRd - MSI) and BRAF V600E mutation. Indeed, the molecular analysis for BRAF gene mutations showed the mutation of the exon 15 (V600E; c.1799 T > A, p. - Val600Glu).

The centenarian was discharged home with in-home geriatric and rehabilitative care and geriatric simultaneous care on a monthly regular basis. Taking into consideration the advanced age and the biological vulnerability, the patient was not considered eligible for adjuvant chemotherapy. Furthermore, the family members were sent to genetic counseling based on the results of the analysis of mismatch repair genes.

After nine months from surgery, the patient lives independently at home with a progressive improvement of functional decline (Barthel Index 70/100, IADL 1/8) and a satisfactory quality of life (EuroQoL 5D 0,71/1).

## Discussion and conclusions

### The Surgeon’s and anesthetist’s clinical standpoint

So far, the clinical management of obstruction of the colon and rectum due to CRC is challenging from a diagnostic and therapeutic standpoint and for the management of septic and oncologic complications [5]. Indeed, few studies had compared theoretical options, mainly dealing with ORCC, since right colectomy and primary anastomosis is considered the best treatment option for ORCC, with a one-step surgical strategy that is generally considered feasible and safe [5].

However, in the last decades CRC has been growingly increased in very old age subjects who are characterized by clinical conditions such as multimorbidity, cognitive impairment, malnutrition and frailty. Similarly, in the present case of a vulnerable centenarian patient with a cancer related obstruction of the right colon, the presence of hemodynamic instability, anemia, hypoalbuminemia and high inflammation had discouraged this recommended surgical approach because for the higher risk of anastomotic leakage, one of the most severe complications after colorectal surgery due to its associated higher morbidity and mortality [20].

The alternative choices recommended by 2017 WSES guidelines if a primary anastomosis is considered unsafe, are terminal ileostomy or self-expanding metal stents [5]. In this specific case the first option was excluded for the increased risk of dehydration or electrolyte imbalance. The other option was excluded because of endoscopic insufflation that might precipitate colonic perforation in a dilated cecum (> 9 cm). Furthermore, the procedure needs to be performed under general anesthesia, and the presence of thick stool may cause fecal impaction within the stent [21]. However, < 5% of all literature on colonic obstruction involves stenting in the proximal colon [22] and scant data are available on stoma, mainly caecostomy, as bring to surgery for ORCC. Recently, a large population-based analysis of ORCC demonstrated that mortality was significantly lower if patients were initially treated with colonic decompression using a minimally invasive procedure as a bridge to surgery, compared with acute resection [23]. This was true especially for older patients with comorbidities [23]. In addition, two Dutch randomized controlled trials were aimed at assessing the performance of stent with emergency surgery, but both trials were prematurely closed due to the high incidence of stent-related complications [24, 25], delivering a decreased indication to stent placement for ORCC from 3.5% in 2009 to 0.5% in 2013. So, this change in decision-making was clinically relevant and in line with the present case report, confirming the need of a more patient-tailored treatment strategy.

As a result, the surgical choice to perform a lateral decompressive caecostomy under local anesthesia was considered the optimal minimal surgical approach, that was also in keeping with patient’s preferences.

Interestingly, even though the current guidelines [5] suggest avoiding the use of surgical caecostomy for the high rate of malfunctioning and complications, this clinical case poses a new attention for its use and indication. Specifically, we could hypothesize that surgical caecostomy might be reserved to a frail and old age patient with obstructive colon cancer, that do not involve the caecum, with dilatation for ileocecal valve continence and with an increased risk of perforation [26]. This first surgical step helps overcoming the major surgery related stress, avoiding a general anesthesia, bridging to the second elective surgery step, with. A key relevant in between time to optimize patient’s clinical conditions and performance status.

The second surgical step with the radical colon surgery was meant to provide a curative intent due to bowel continuity recanalization, the avoidance of the ostomies and its burdensome impact on quality of life [27].

Furthermore, concern has been growing over the last decade regarding whether the aging brain is more vulnerable to anesthesia, because older surgical patients frequently experience a postoperative deterioration in cognitive function. However, the impact of anesthesia on frail patient’s recovery from surgery and its relationship with geriatric syndromes is far to be completely explained. Neurophysiological and anatomical changes are relevant to understand these complex relations in the ageing brain, but several factors are involved, including surgical stress, inflammation, pain, comorbidities and the phenotypic trajectory of a patient’s cognitive decline with age [28].

In line with other guideline statements specifically dedicated to elders’ anaesthesia and peri-operative care [17–19], NICE guidelines address the importance of depth of anaesthesia monitor during any type of general anaesthesia in patients at higher risk of adverse outcomes [29, 30]. Moreover, in 2018, ERAS® Society strongly recommended the use of short-acting anesthetics, intraoperative cerebral monitoring and monitoring of the level and complete reversal of neuromuscular block in order to improve functional recovery and to reduce the risk for postoperative delirium [31].

In conclusion, the improved surgical techniques and the advancements in anesthesiology made this surgery feasible for the majority of older patients, with no definitive consensus about which is the optimal surgical management for older people admitted to the emergency department with diagnosis of complicated CRC. Similarly, a paucity of studies evaluated early and late outcomes in cohort of patients aged over 90 [3, 32, 33]. To the best of our knowledge, this is the first report of a successful surgery with curative intent for ascending colon cancer in a centenarian with a postoperative follow-up of nine months. A two-steps colon surgery, in the form of a primary cecostomy and a second step right colectomy with ileo-colic anastomosis in an oldest old patient has never been reported so far. This surgical approach, showed to be safe and feasible in a very old age patient in term of prolonged survival and adequate quality of life, indicating the need for dedicated surgical algorithms for older patients tailored on the basis of specific cancer related issues and patient’s biological status and preferences.

### The Geriatrician’s clinical standpoint

Older patients represent a challenge for surgery [34], having an excess of morbidity and mortality, that is the result of the complex interaction between clinical and biological features of aging. Chronological age is a very insufficient proxy of a senior patient’s vulnerability. The systematic assessment of CGA is the gold standard to deliver patient-tailored multidisciplinary interventions.

Although the American College of Surgeons and the American Geriatrics Society recommended a preoperative frailty assessment for all old-age patients, who are candidate for surgical procedures [35], screening for frailty is rarely applied in routine clinical practice. Namely, CGA assessment is performed by 6% of the surgeons and overall, the multidisciplinary approach with geriatricians is rather uncommon among surgeons [36].

Our report describes the successful surgeon and geriatrician co-management model moving a step forward sin the delivering of [37] of effective treatments in oldest old cancer patient.

Frailty, sarcopenia, poor functional status, cognitive impairment and multimorbidity are clinical factors that can affect a patient clinical trajectory, being independent risk factors for major morbidity, mortality, increased length of stay and institutionalization. In line with that, the pre-surgery assessment of frailty should be always advocated for overall risk stratification and for the identification of potentially modifiable factors [38].

However, in general practice, there is the erroneous tendency at looking at frailty as a condition for excluding patients from active treatment or, at best, for justifying a lower intensity care. Actually, for geriatrician the detection of frailty should instead represent the entry point for a more in-depth analysis aiming at identifying the causes of individual’s increased vulnerability and implementing a person-tailored intervention plan [39]. Moreover, the case of the management of a centenarian patient requiring emergency surgery poses challenging questions also for geriatric medicine, since the complex biology of oldest old had not completely understood so far. So, the geriatrician is called to interpret the results of a multidimensional assessment and encourage a clinical process that shift the attention to the intrinsic capacity of an individual rather than deficits and abnormalities [40].

Starting from this background, the preoperative period has to be recognized as a window of opportunity to further improve patient outcomes. Based on the preoperative identification of these features and geriatric syndromes, the application of pre-habilitation programs is an interesting attempt to revert older adults’ frailty and to enhance resilience prior to a surgical treatment, choosing tailored intervention based on a patient’s impaired domains.

**Fig. 2:**
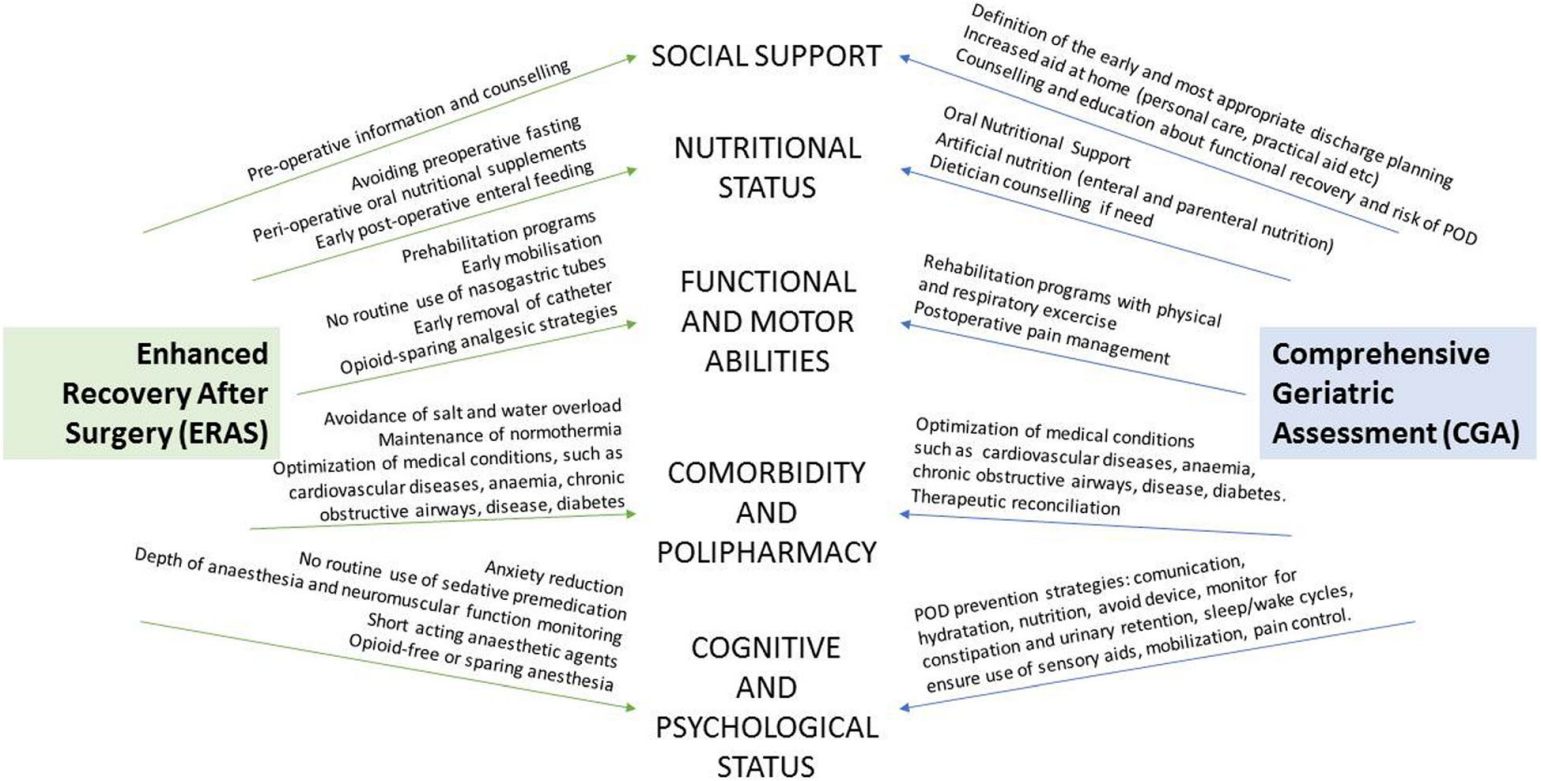
A brief summary of the main key points shared by ERAS surgical approach and geriatric approach based on Comprehensive Geriatric Assessment (CGA). Abbreviations: CGA Comprehensive Geriatric Assessment; ERAS Enhanced Recovery after surgery; POD postoperative delirium

Furthermore, this case report is an example on how very old ages and clinical complexity would mostly benefit from the Enhanced Recovery After Surgery (ERAS) protocols, under the supervision of a multidisciplinary team. This fast-track protocols, include evidence-based items designed to reduce perioperative stress, maintaining postoperative physiological function and accelerating recovery after surgery [31, 41]. From a geriatric perspective, this multimodal stress-minimizing approach shares many key concepts with geriatric issues (Fig. 2), such as minimizing organ dysfunction through avoidance of device as catheter, drains, nasogastric tube, early enteral feeding and the promotion of patient’s early mobilization in the frame of multimodal care [41] as recently identified by a retrospective review [42].

A 2014 systematic review found that, even though ERAS programs can be safely applied in older patients to reduce complications and shorten length of hospital stay, further studies are needed to assess the clinical utility and effectiveness in very old patients [41]. In line with that, it could be hypothesized that the geriatrician could be in charge of the modulation of each ERAS items on the basis of the patients’ individual biological and functional reserves and based on the systematic CGA assessment and related interventions. This combined approach could be of key relevance for tailoring perioperative protocols in older adults and for maximizing their fitness for surgery.

So, this clinical case offers the opportunity to reflect upon the construction and validation of a multidisciplinary approach that strongly incorporate the presence and expertise of geriatrician in the treatment planning of older cancer patient, in order to bring the perspective of focusing on functional preservation and quality of life as the outmost important treatment goals.

In conclusion, this surgical case deals with the “real world” demographic shift and ‘the biological heterogeneity of the older population, addressing the need for revision of previous models of care that considered the chronological age as the single criterion for selecting cancer for surgery.

the biological complexity of older patients requires changes in their management and the revision of anesthetic and surgical techniques, in light of the clinical complexity of very old age patients. In addition, multidisciplinary teams need all to aim at preserving quality of life and autonomy in daily living rather than long-term survival, since it is of the utmost importance for many older patients.

Multidisciplinary teams including geriatricians along with surgeons and anesthesiologists have been established so far in the orthogeriatric field, but there are very scant evidence on the effectiveness of such teams in oncological surgery [43–46]. Yet, we predict that, once established, such teams will successfully improve many clinical outcomes associated with solid tumor and their surgery, improving patient’s functional status and quality of life.

as older cancer patients are rarely enrolled in clinical trial, with scant evidence-based result for the oldest old, there is an urgent need to carry out high quality research into new models of care, pre-operative risk stratification and optimization [47, 48]. Moreover, anesthesiologists, surgeons and geriatricians should receive specific training in the assessment and management of older surgical patients, as an key relevant step for the optimization of their care.

## Data Availability

The data are completely available in this article.

## Abbreviations

BIS: Bi-spectral index
CDT: Clock Drawing Test Shulman
CGA: Comprehensive Geriatric Assessment
CI: Co-morbidity Index
CIRS: Cumulative Illness Rating Scale
CRC: Colorectal cancer
ERAS: Enhanced Recovery After Surgery
FI: Frailty Index
GDS: Geriatric Depression Scale
I-ADL: Instrumental Activities of Daily Living
MAP: Mean Arterial Pressure
MMSE: Mini Mental State Examination
MNA: Mini Nutritional Assessment
NICE: The National Institute for Health and Care Excellence
ORCC: Obstructive right colon carcinoma
SI: Illness Severity Index
TOF: Train of four
WSES: World Society of Emergency Surgery
